# Mechanism and Design Analysis of Articulated Ankle Foot Orthoses for Drop-Foot

**DOI:** 10.1155/2014/867869

**Published:** 2014-04-30

**Authors:** Morshed Alam, Imtiaz Ahmed Choudhury, Azuddin Bin Mamat

**Affiliations:** Manufacturing System Integration, Department of Mechanical Engineering, University of Malaya, 50603 Kuala Lumpur, Malaysia

## Abstract

Robotic technologies are being employed increasingly in the treatment of lower limb disabilities. Individuals suffering from stroke and other neurological disorders often experience inadequate dorsiflexion during swing phase of the gait cycle due to dorsiflexor muscle weakness. This type of pathological gait, mostly known as drop-foot gait, has two major complications, foot-slap during loading response and toe-drag during swing. Ankle foot orthotic (AFO) devices are mostly prescribed to resolve these complications. Existing AFOs are designed with or without articulated joint with various motion control elements like springs, dampers, four-bar mechanism, series elastic actuator, and so forth. This paper examines various AFO designs for drop-foot, discusses the mechanism, and identifies limitations and remaining design challenges. Along with two commercially available AFOs some designs possess promising prospective to be used as daily-wear device. However, the design and mechanism of AFO must ensure compactness, light weight, low noise, and high efficiency. These entailments present significant engineering challenges to develop a new design with wide consumer adoption.

## 1. Introduction

**Figure 1:**
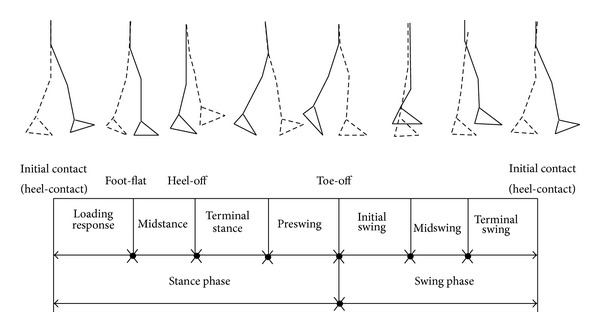
Different phases of normal gait cycle.

Stroke is considered as the leading cause of disability throughout the world [1]. Individuals suffering from stroke and other neurological disorders have reduced walking capacity, which has a great impact on daily life [2]. Various damages in neuromuscular system, presence of spasticity, contracture, and weakness can also result in walking speed reduction, elevation in energy cost, and an increased risk of falling. The main cause of musculoskeletal impairment is the weakness of plantar flexor and dorsiflexor muscles. Plantar flexor muscle weakness would result in reduction of push-off power and elevation in energy cost of patient as most of the power in walking is generated during ankle push-off. Individuals with dorsal muscle weakness are not capable of lifting the foot adequately in midswing due to insufficient dorsiflexion; it results in toe-dragging, lowering walking speed, shortening of step length, elevation in walking metabolism, and high risk of tripping. “Foot-slap” and toe-dragging are the major complications of the patients having dorsiflexor muscle weakness. “Foot-slap” is the uncontrolled and rapid strike of foot on the ground producing distinctive sound at heel strike and “toe-drag” means dragging of forefoot during walking due to inadequate ground clearance during swing phase of the gait cycle [3].  Figure 1 shows different phases and terms of normal gait cycle. Other than stroke, people of any age could suffer from muscle weakness because of trauma, brain injury, spinal cord injury, muscular dystrophy, and so forth [4, 5].

There are a number of treatments for ankle foot disabilities such as surgical, therapeutic, or orthotic. Applying functional-electrical stimulation (FES) is an active approach to the drop-foot gait treatment [6]. It is a technique that uses electrical current to contract damaged muscles. Besides FES, this technique has different names such as electrical stimulation and functional neuromuscular stimulation (FNS). However, all of them have the same goal to stimulate damaged muscle contraction and enhance functionality. It is applied to the common peroneal (CP) nerve during the swing phase of the gait cycle, which stimulates the functionality of the dorsiflexor muscles [7]. Through this stimulation the ankle can be flexed beyond neutral angle, which helps the ankle foot complex maintain toe-clearance during the swing phase [8]. However, activated muscle mass by FES is the fraction of available muscles resulting in less effectiveness for drop-foot prevention, which is a disadvantage of this approach [9].

Among these approaches, orthotic treatment is the most common practice. Moreover, plantar flexor muscles are not frequently affected; that is why most of the ankle foot orthotic devices are designed for drop-foot prevention [10]. In general, there are three types of ankle foot orthotic (AFO) devices: passive devices, semiactive devices, and active devices. Passive AFO device is not comprised of any electrical or electronic elements or any power sources. It may be comprised of mechanical elements like dampers or springs to control the motion of ankle-foot complex. Semiactive AFO devices are capable of varying flexibility of the ankle joint by using computer control. Active AFOs contain onboard power source, control system, sensors, and actuators. Among these devices, passive AFO is the most popular daily-wear device due to its compactness, durability, and simplicity of the design. Active and semiactive AFOs have the limited usage only for rehabilitation purpose due to the need of improvement of actuator weight, portable power supply, and general control strategy.

We have found two types of literatures on ankle foot orthotic devices: one type focusing on design and construction and another type focusing on the evaluation of gait wearing AFO. Two recent reviews are found: one describing possibilities and challenges of powered ankle foot orthotic devices [11] and another one reporting challenges and state of the art of lower extremity exoskeleton and active orthotic devices [12]. The purpose of this paper is to review the engineering design of AFO with articulated ankle joint, developed in recent years for “drop-foot” treatment. We will limit our focus on design elements, design considerations, and working mechanism of the devices. The study is arranged as follows: Section 2 describes construction elements of AFO, Section 3 provides design considerations of AFO, Section 4 presents various designs and mechanisms of articulated AFO, and Section 5 gives overall discussion on these designs.

## 2. Construction Elements of AFO

Passive ankle foot orthotic (AFO) devices do not consist of any electronic control element other than mechanical elements like spring or damper to control the ankle joint motion during gait. These devices are of two types: articulated and nonarticulated. Nonarticulated AFOs are usually single piece, fabricated out of lightweight thermoformable or thermoplastic materials, and encompass the dorsal part of the leg and bottom of the foot. The designs of these AFOs vary from highly rigid to flexible. AFO with rigid ankle holds the ankle in a fixed position, restricts the plantarflexion mobility completely, and maintains clearance between forefoot and ground. However, if the AFO is too rigid it causes excessive knee flexion moment during loading response that results in walking instability. Posterior leaf spring AFO is a semirigid plastic AFO that assists push-off during preswing and prevents drop-foot. Features and characteristics of these AFOs depend on material and geometrical shape [13–17]. Carbon fiber AFO is also a spring type AFO and it is capable of improving pathological gait significantly by storing energy during deformation and augmenting push-off during preswing. Different researches have shown that carbon fiber AFO can decrease the energy expenditure of the impaired patient [18, 19]. However, though nonarticulated passive AFO improves pathological gait to some extent, it restricts some movements having functional benefit.

Passive articulated AFOs are designed combining light-weight thermoplastic or carbon composite shells and articulated joints. There are many different designs of articulated joints with a variety of hinges, flexion stops, and stiffness control elements like spring, oil damper, one-way friction clutch, and so forth. Commercial hinge joints like Tamarack flexure joint and Klenzak ankle joint with pin or spring are used to control the motion of ankle in sagittal plane [20]. AFOs with commercial joints and mechanical stops are capable of preventing drop-foot successfully by providing dorsiflexion assisting force or locking the ankle in a suitable position, but these AFOs also inhibit other normal movement of the ankle. To overcome this problem researchers have introduced different motion control elements for providing normal gait motion. Articulated AFOs with those elements can provide adjustability of initial ankle angle and joint stiffness, better motion control of foot, assistive force in dorsiflexion direction, resistive force in plantarflexion direction, and desirable range of motion of the ankle joint. There are some innovative passive AFOs that utilize the energy from gait to provide assistive motion. These AFOs are called power harvesting AFOs and some pneumatic components like bellow pump, passive pneumatic element, and so forth are used for locking the foot or providing assistive torque.

The motion control of passive AFOs is limited by passive elements; those are not capable of adapting to changing environment and have limited functionality for disabled persons. Active AFOs possess the ability to interact with the walking environment and act accordingly. These types of AFOs are comprised of electronic control system, actuator, tethered or untethered power system, and stiffness control element like magnetorheological brake for better control of ankle motion. The control system usually includes components like force sensor, angle measuring sensor, accelerometer, and microprocessor.

Some motion control elements that are successfully used in different ankle foot orthotic devices are described in the following section.

*Spring.* A study by Palmer has shown that ankle function can be considered as a linear torsional spring during controlled plantarflexion [21]. In most of the articulated AFOs, springs are used to provide controlled plantarflexion during loading response to prevent foot-slap and in some cases to assist dorsiflexion. In conventional AFOs, hinge joints are comprised of springs to resist or assist ankle movement along with various types of stops. Yamamoto et al. [22] developed an articulated AFO called “dorsiflexion assist controlled by spring” (DACS) AFO for hemiplegic patients to prevent drop-foot. A spring in the posterior part of the tibial upright of the AFO generates plantarflexion resisting moment during heel strike and prevents foot-slap. It is capable of providing 2–17 Nm dorsiflexion assisting moments per 10 degrees of plantarflexion. Stiffness of the 300 g DACS AFO can be altered by changing springs of different coefficients. 

*Series Elastic Actuator.* SEA is a good resolution for resembling torque sources at joints. An elastic element is employed in series with a DC motor to construct this actuator. By changing the deflection of the elastic element, output force can be changed. A control system is used to actuate the motor for the desired output force. This actuator has extremely low impedance, good force control bandwidth, shock tolerance, low friction, and high force fidelity. Moreover, it can provide both plantarflexion and dorsiflexion assistance and is also capable of controlling ankle joint impedance. These characteristics are favorable for various applications such as adaptive suspensions, robotic arm, exoskeleton, legged robots, and orthotic devices [23, 24]. 

*Magnetorheological Fluid.* Magnetorheological (MR) fluid is a solution carrying magnetic metal particle in a carrier fluid, usually oil. The viscosity of this fluid changes rapidly when a magnetic field is applied. This change is demonstrated by the increase of yield stress that develops with the applied field. This material can provide quiet, simple, and rapid response between mechanical and electrical system interfaces [25]. We have found two AFOs that use MR fluid for developing brake and damper. 

*Passive Pneumatic Element.* Kawamura et al. [26] developed a passive mechanical element, which has variable elasticity and viscosity. The material of this element is soft and light in weight and the element itself is small in size. It is possible to alter the mechanical impedance of this element by adjusting the vacuum pressure applied to it. These characteristics make the passive pneumatic element more convenient over other active elements of wearable robots like electromagnetic brake, magnetorheological brake or electrorheological brake, and so forth. 

*Frictional Clutch.* We have found one ankle joint of an AFO using one-way frictional clutch. This clutch allows free motion in one direction and constant resistance in the other direction. This clutch is also used in knee brace. The material of the element is selected in such a way that it can hold human body weight. 

*Oil Damper.* Oil damper is one kind of hydraulic shock absorber which uses hydraulic resistance. Yamamoto et al. [27] developed a small, lightweight hydraulic oil damper to provide plantarflexion resistive torque on the ankle joint. Oil damper is capable of absorbing shock during heel strike and providing damping during loading response. 

*Artificial Pneumatic Muscle.* Artificial muscles are pneumatic devices controlled by compressed air filling pneumatic tubing. Physician Joseph L. McKibben invented the first McKibben pneumatic muscle in the 1950s to develop pneumatic arm orthotics. Such devices possess high level of functional similarity with human skeletal muscle and high power to weight ratio [28]. Ferris et al. [29, 30] developed a tethered powered ankle foot orthosis with McKibben pneumatic muscles to study human walking and also for rehabilitation purpose. Unlike other ankle foot orthosis this device can provide both plantarflexion and dorsiflexion torque. 

*Shape Memory Alloys.* Esfahani [31] developed a conceptual design of articulated AFO with shape memory alloy (SMA) actuator. SMA material possesses the ability to undergo seemingly large plastic strain, subjected to heat or other stress related alterations, and afterward retrieve the strain when heat or stress is withdrawn. SMA actuators have high power to weight ratio, which allows it to be used in designing compact devices. Other advantages such as phase transformation sensibility, low driving voltage, and noiseless operation could resolve many issues to develop an effective AFO. However, slow response and mechanical inefficiency leave strong technical challenges in its implementation [31].

## 3. Design Considerations of AFO

Orthotic device design requires consideration of dynamics of the original limb, which makes it more challenging than designing prosthetic devices. For the treatment of drop-foot, an ideal AFO should compensate dorsiflexor muscle weakness by preventing unwanted plantarflexion motion of ankle without affecting normal movement. AFO should provide moderate resistance during loading response to prevent foot-slap, no resistance during stance for free ankle motion, and large resistance to plantarflexion during swing phase to prevent drop-foot.

According to Yamamoto et al. [20, 27] an ankle foot orthosis should have an articulated ankle joint. There should be provision of initial angle adjustment in a range from 0 to 8°. In dorsiflexion direction, the joint should not provide any resistive moment and the range of motion should be more than 30° from initial ankle angle. In plantarflexion direction, AFO should generate resistive moment and it should be adjustable in a range from 5 to 20 Nm per 10° of plantarflexion. The required resistive moment during swing phase is five times more than the resistive moment required in loading response.

Stiffness of ankle joint is to be maintained properly; otherwise it could hamper functional activities of the patients. If an AFO is less stiff, plantarflexion resisting moment will not be sufficient enough to hold the foot and keep clearance during swing. Conversely, if an AFO is excessively stiff, it will resist the ankle plantarflexion at initial contact resulting in a delay in loading response and excessive knee flexion motion and moment. AFO with high stiffness also obstructs ankle dorsiflexion during stance which causes instability and reduction in walking speed. An ankle foot orthosis with excessive stiffness can also delay the rehabilitation of patients with neurological damage.

The ankle trajectory varies largely in stair or inclined surface walking from level walking. Different phases of gait cycle differ in both ascending and descending inclined ground surfaces. During stair ascending the major difference from level walking gait is found in stance phase and late swing; throughout the stance phase the foot remains dorsiflexed and at late swing to avoid the edge of stair the foot is dorsiflexed. During stair descend, at loading response the toe strikes the ground before heel, and a large dorsiflexion is found during stance phase to allow the downward and forward movement of the body. Thus an idle AFO design should possess adjustability to respond to the ground variation [32].

An AFO should be compact in size and light in weight to facilitate daily life use. The powered AFO should have compact actuator, portable power source and provision of adjustability with the change of patient's condition.  Table 1 presents the summary of various features of articulated AFOs.

## 4. Design and Mechanism of AFO

### 4.1. Dream Brace

ORTHO Incorporation, Japan, first developed “dream brace,” whose function is to provide ankle movement according to the gait cycle. The active element for the innovative mechanism of the articulated joint used in this AFO is a one-way frictional bearing clutch. This joint is of two types; type A and type B. Type A joint has a dial rock mechanism with three different angle settings to adjust plantarflexion at position of angle 13°, 38°, or −7° (for knee brace), and type B joint has free plantarflexion. Dorsiflexion is maximum 100° and same for both types of joints. Resistance strength of the frictional bearing is fixed and resistance torque can be selected from the chart provided by the manufacturer for different sizes. The weight of the brace is approximately 350 g and the material used for this joint is SUS304 stainless Steel.

During heel strike at initial contact, the friction of the dream joint dampens the foot-slap by providing resistance to planter flexion. Unlike spring-loaded AFO the resistance torque of the joint does not increase as the foot approaches the ground. During stance phase the body moves forward and the ankle joint allows free dorsiflexion motion as there is no frictional resistance in this direction. During swing phase the joint holds the foot to ensure clearance between toe and ground [33, 34]. No published literature was found describing clinical assessment of the AFO joint.

### 4.2. AFO with Oil Damper

**Figure 2:**
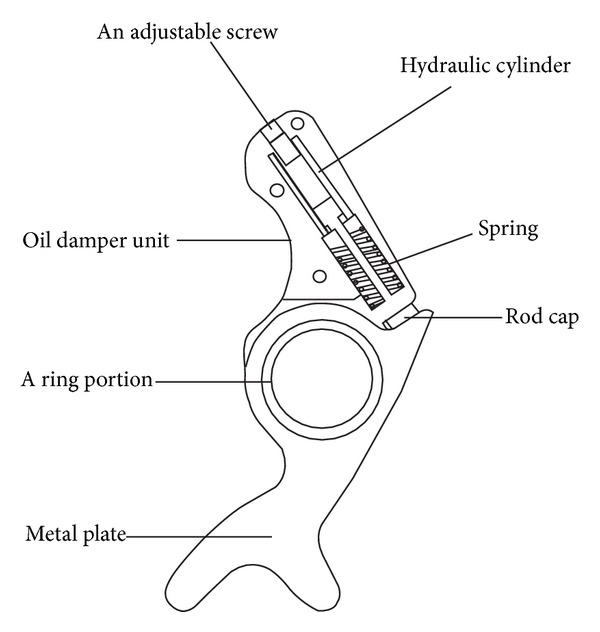
A schematic diagram of the ankle joint of AFO with oil damper [34].

Kawamura Gishi Co. Ltd., Japan, produces “Gait solution orthosis” that features an articulated ankle joint with oil damper and a spring. Yamamoto et al. [27] developed this ankle foot orthosis based on some specifications from previous study [35]. In this AFO, a specially designed ankle joint with cam mechanism is attached to the lateral side of the ankle. Components of the articulated joint are showed in Figure 2.

During loading response, with the plantarflexion movement of the ankle, the piston of the oil damper is moved upward forcing oil through the orifices of the cylinder wall. The orifice restricts the flow of the oil and provides resistive plantarflexion moment which can be varied in a range from 5 to 20 Nm per 10° of plantarflexion by varying the diameter of the orifice with the help of an adjustment screw at the top of the oil damper. The cam mechanism converts the rotational movement of the ankle joint to linear compression of the oil damper. The oil damper restricts only plantarflexion motion and does not assist dorsiflexion. During stance phase, a small spring assists the ankle to move freely in dorsiflexion direction and helps the piston return to the initial point and oil to the cylinder through check valve. This spring also ensures toe-clearance during swing phase. A rod cap at the bottom of the piston rod is used to set the initial angle in between 0° and 8°, which is necessary to ensure stability in stance phase [27, 36]. Adjustability of ankle joint stiffness and initial ankle angle are the two advantageous features of this AFO that are important for controlling body alignment while walking.

### 4.3. Power Harvesting AFO

**Figure 3:**
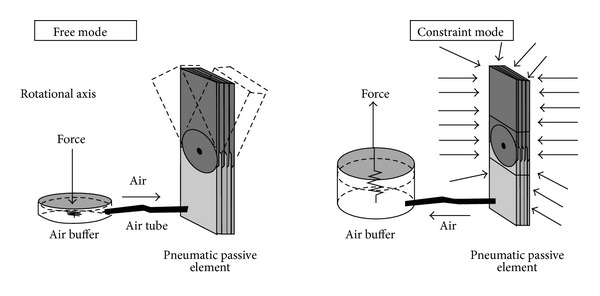
Free and constraint mode of passive pneumatic element [37].

Hirai et al. [37] proposed a new design of passive AFO with pneumatic passive element, which is made of thin laminated sheets of polystyrene in an airtight chamber. This element was modified with a rotational axis and placed at the axis of rotation of ankle to control motion. The vacuum pressure inside the chamber influences the frictional force between the laminated sheets. An air buffer functioning similar to a pump is attached under the sole, controls the air flow to the passive element chamber, and alters the vacuum pressure to change the constraint force on the elements. During loading response, the strong constraint torque of the joint prevents foot-slap. During midstance to toe-off, the buffer is compressed due to body weight and air flows to the passive element causing the sheets of the element to rotate freely around the axis and allowing the ankle joint to move without any restriction. During swing, air comes out from the element causing the thin sheets to stick together to prevent drop-foot (Figure 3). By adjusting the constraint force of the pneumatic element, this AFO can imitate the functional characteristics of other AFOs. This AFO is favorable for patients because of its light weight and compactness.

**Figure 4:**
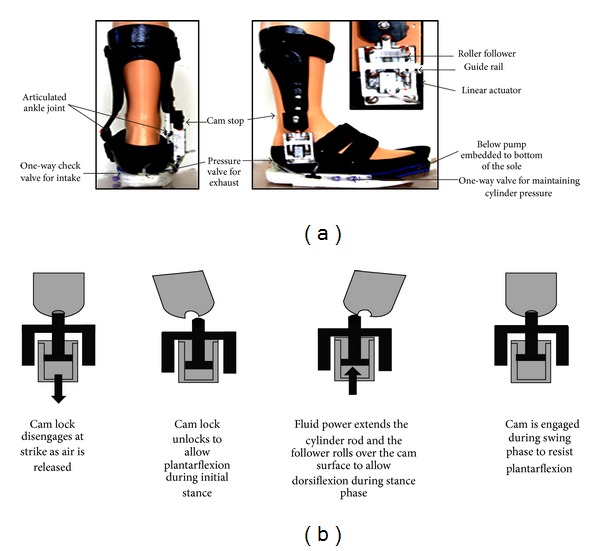
(a) Posterior and lateral view of a power harvesting AFO (b) engaging and disengaging of cam lock during gait cycle [10].

A novel design of AFO, which can harvest energy during middle to late stance and ensure toe-clearance by means of locking the ankle in neutral position during swing, was developed by Chin et al. [10]. The design is comprised of two sections, tibial upright and foot, fabricated from carbon fiber composite material. These two sections are attached with each other with a conventional hinge joint. The control system of the articulated joint integrates a cam lock mechanism, a linear actuator, and a pneumatic circuit. A stationary cam is added to the lateral side of the tibial upright and the linear actuator is attached to the foot section (Figure 4(a)). The actuator mechanism is comprised of a linear cylinder with spring return, a follower with small rollers, and a guide rail housing for the follower. The minimum pressure needed to move the cylinder rod is 120 KPa. A pneumatic circuit is located in the plantar surface of the foot section. The circuit is comprised of a bellow pump with 4.5 cm outside diameter, valves, and tubing. The size, shape, and design of the bellow are determined in such a way that it can achieve pressure above 150 KPa and generate around 10 KW power per gait cycle [38]. The bellow pump is placed under 2nd and 3rd metatarsal heads. This placement provides best possible pressure generation and optimal timing for release valve and actuator activation. The release valve discharges compressed air of the actuator cylinder into the atmosphere. During heel strike this valve is activated and the spring in the cylinder pulls back the actuator rod and unlocks the cam lock to allow free movement of the ankle. During midstance to late stance the release valve is closed, the weight of the body compresses the below pump, and the harvested fluid power extends the cylinder rod and follower. The design of the cam allows the follower to roll over the cam surface to permit ankle dorsiflexion. During late stance, due to plantarflexion of the ankle the follower rolls into the locking position and locks the ankle in neutral position to prevent foot drop in swing phase (Figure 4(b)). The outsole prototype of the AFO is compact but cosmetically not attractive and clinical assessment is not done.

**Figure 5:**
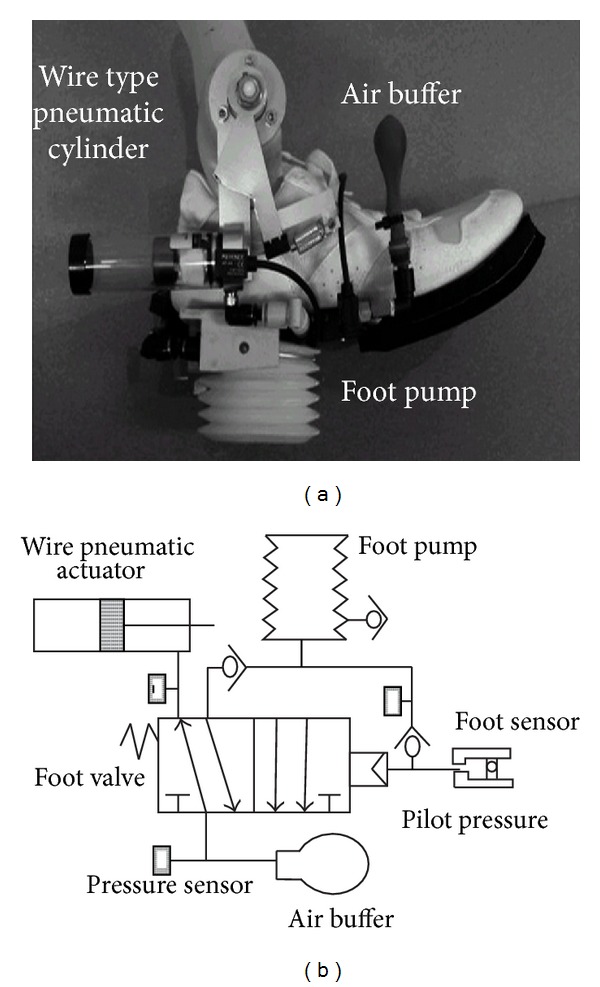
(a) Different components and (b) pneumatic driving circuit of a power harvesting AFO [39].

Takaiwa and Noritsugu [39] from Okayama University, Japan, developed a prototype of a portable pneumatic power harvesting ankle foot orthosis that can raise the dorsiflexion angle by 20° in swing phase by providing dorsiflexion assisting moment (Figure 5(a)). The design includes a commercially available AFO (dream brace), a wire type pneumatic actuator cylinder, and a pneumatic circuit in the plantar surface of the foot (Figure 5(b)). The pneumatic actuator is affixed to the articulated joint of AFO with a moment arm. It acts as a driving actuator with high power/weight ratio and it can be used in a narrow space as it is driven by a wire instead of piston rod. A balloon is inserted into the cylinder which acts as a seal and the wire is connected to the piston inside the cylinder. The pneumatic circuit is comprised of a bellow pump placed under the heel, a mechanical sensor located under the toe, a five-port pilot valve at the middle of the shoe bottom to switch the flow direction, and an air buffer. The mechanical sensor is connected to the pilot valve, which changes the flow direction by lowering pilot pressure. During stance period, the bellow pump is compressed and at a certain pressure (about 60 KPa) the pilot valve is actuated and compressed air starts to accumulate in the air buffer. At the late stance the toe steps on the mechanical switch and then in swing phase at atmospheric pressure the pilot valve switches the accumulated air flow from the air buffer to the actuator cylinder. As the cylinder pressure rises, the piston pulls the moment arm to generate dorsiflexion ankle moment. The AFO can produce small dorsiflexion assisting moment around the ankle. Moreover, the bulky nature of the AFO is not suitable for daily use.

### 4.4. AFO Using Magnetorheological Fluid

**Figure 6:**
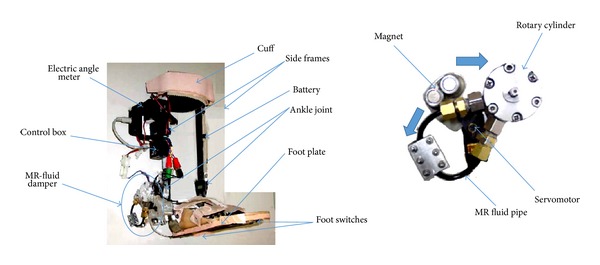
The intelligent AFO with MR fluid damper [40].

Researchers from Osaka University, Japan, developed a prototype of intelligent AFO that has an ankle joint with adjustable resistance torque [40]. The AFO is comprised of a pair of frame and a foot plate made of carbon fiber polymer, cuff, and a variable resistance ankle joint system using magnetorheological fluid (MR). The ankle joint components are a rotary cylinder, a servometer, and a permanent magnet. The rotary cylinder of two chambers, separated by a vane, is filled with MR fluid. This fluid flows from one chamber to another through a polypropylene pipe (Figure 6). The resistance torque of the rotary cylinder is changed by varying the viscosity of the MR fluid which is done by changing the distance between the magnet and the MR fluid flowing pipe by means of the servomotor. The control system of the articulated joint includes an electric angle meter, force sensor affixed to the sole at heel and forefoot region, a control box with microprocessor, and a battery. The angle meter and foot sensors are used to identify the phase of the gait, while the angle meter measures the shank angle to the vertical, and the foot sensor detects the ground contact. The authors measured resistance torque with the change of angular velocity for four different viscous resistance modes and set default control rules for a healthy volunteer. From mode 1 to mode 4 the resistance torque changes from least to the greatest. According to the default control rule, at initial strike the resistance torque is set to mode 2 that provides moderate resistance to plantarflexion movement of the ankle to prevent foot-slap. During stance phase, the resistance torque is set to the most flexible mode to allow free movement of the ankle. At late stance, while the shank angle is 10° to the vertical, the most rigid mode is set to prevent foot drop in swing phase. Compared to other active AFOs, this AFO requires smaller power unit but the weight of the prototype is to be reduced for making it a daily-wear device. Moreover, the control settings require an expert for adjustment.

**Figure 7:**
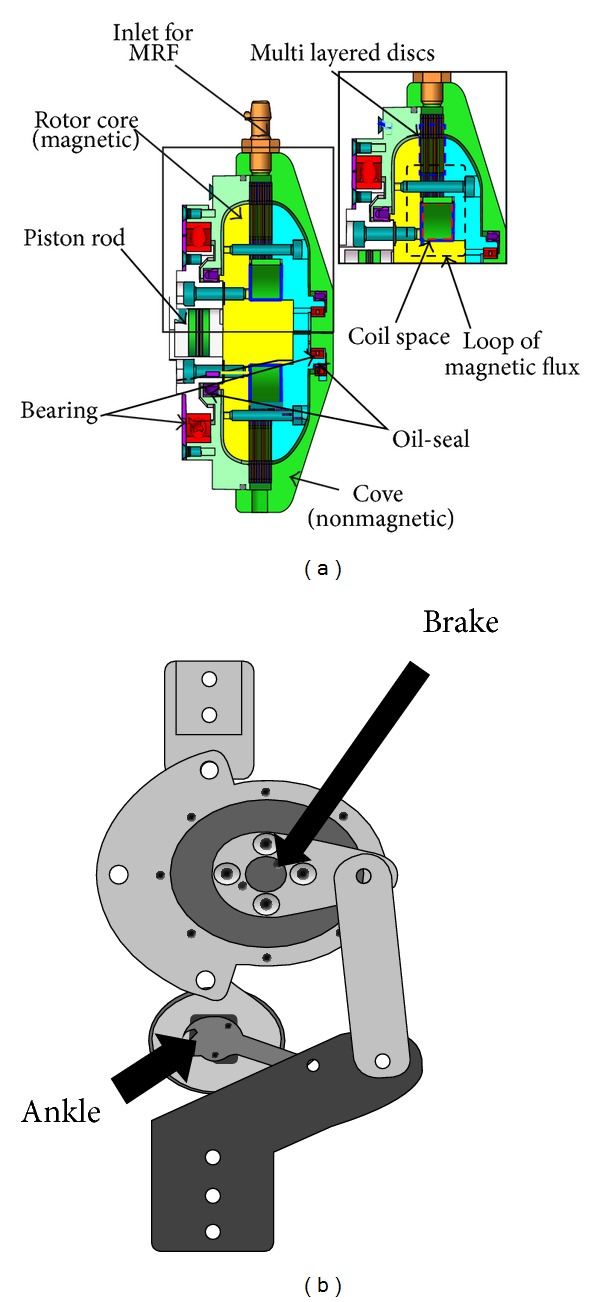
(a) Schematic of MR brake cross section of MR brake, (b) linkage mechanism [41].

Furusho et al. [25] developed an active AFO that uses shear-type compact MR brake to control the ankle joint movement. The brake system is comprised of a nonmagnetic housing which is fixed to the orthotic device, disc type rotor attached to a shaft (ankle axis), stator attached to the housing, and a coil around the shaft (Figure 7(a)). The gap between the disks is filled with MR fluid. A piston mechanism is used to prevent fluid leakage. When current passes through the coil, a magnetic field is created around the coil which acts on the MR fluid. The modulation of viscosity develops high shear stress and brake torque. The system is able to produce maximum 11.8 Nm resistive torque. A linkage mechanism is utilized to amplify the torque and the amplified torque is 24 Nm (Figure 7(b)). The control system of the AFO involves a potentiometer at the ankle to measure the angle, a bending moment sensor in the lateral part of the shank of the AFO, a six-axis force torque sensor attached to the middle of the bottom of the foot plate of the AFO to measure the ground reaction force, and a computer. The control algorithm divides the gait motion into four different states. During heel strike, to absorb the shock and prevent foot-slap, braking torque is provided in proportion to the ankle's angular velocity. During stance phase no braking torque is provided to allow free motion and in swing phase to ensure toe-clearance the system provides enough braking torque. MR brake consumes 3–6 W power and the weight of the AFO is 1.6 Kg.

Kikuchi et al. [41] improved the previous AFO with MR brake by developing a 5 Nm class compact magnetorheological fluid brake (CMRFB). This new AFO is less in weight (990 g) compared to the previous prototype and the control system is improved. The new CMRFB can produce about 10 Nm torque, which is sufficient to prevent foot drop but not sufficient to control plantarflexion during loading response. A spring unit, on the ankle joint, is introduced to the new prototype to provide a controlled plantarflexion movement of the ankle during loading response. A liver mechanism pushes the spring to restrict plantarflexion movement of the ankle. In control system, foot sensor switches are replaced by an accelerometer which is attached to strut of the AFO. The new specifications and control system of the AFO resulted in good controllability and high torque to work ratio.

**Figure 8:**
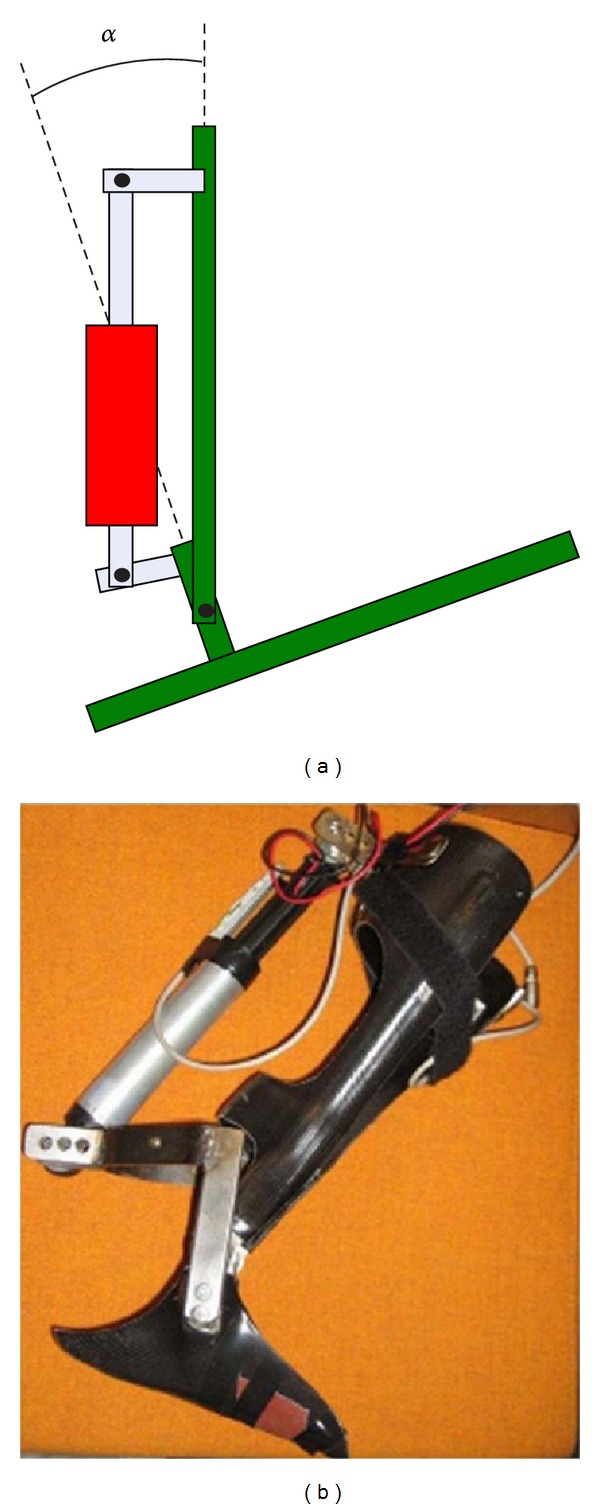
Halmstad University AFO with MR fluid damper; here *α* is the adjustable ankle angle [32].

Svensson and Holmberg [32] developed an AFO that has adjustable features for different ground conditions, such as ascending or descending stairs or inclined surface. This orthotic device also used magnetorheological damper to control the ankle motion (Figure 8). It is constructed of composite material with one steel joint on each side of the ankle. The degree of freedom of the device is eight degrees in plantarflexion direction and twenty-six degrees in dorsiflexion direction. The linear damper is affixed to the posterior side of the leg with a linkage system. The control system consists of a 40 MHz PIC18F microprocessor, an angle measuring sensor with 10-bit AD converter, and low pass filter, and all electronics are embedded into a small box attached to the AFO. The device also is comprised of a specialfeature, a Bluetooth unit for sending data to a PC, to analyze the system performance. The control system uses an eight-bit pulse width modulated signal to adjust the damper current according to the different phases of the gait. The device has four states of damping.

- Damp: moderate damping during foot down to prevent foot-slap.
- Free: very small or no damping during stance phase to allow free motion.
- Lock: high damping to lock the ankle during swing phase.
- Free down: very small or no damping during swing and stance to allow free motion.

The ground condition or gait situation, ascending or descending inclined surface or stair, sets the sequence of these states. Switching between the states depends on the ankle's angle. During level walking the AFO provides moderate damping at heel strike, free motion in stance phase, and high damping to lock the ankle motion in swing phase. Stair ascending involves only two states: high damping state when the foot is in the air and free state when the front part of the foot contacts the steps of the stair. During stair descending, damping state is not involved and free down state is employed to allow the toes to point down when the toes are in the air. A test with three healthy participants walking on a treadmill at various inclinations ascending and descending stairs authenticated the success of the design and control algorithm.

### 4.5. AFO with Four-Bar Mechanism

**Figure 9:**
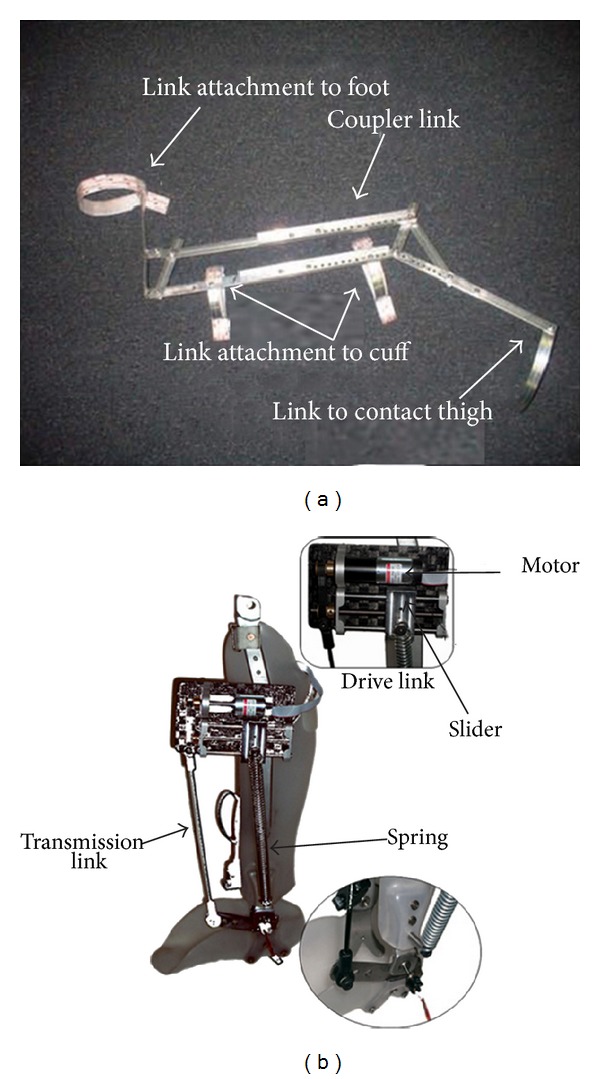
(a) Passive AFO with four-bar mechanism [43]. (b) An AFO with insertion point eccentricity control [9].

Berkelman et al. [43] developed a novel ankle foot orthosis design based on passive four-bar linkage mechanism (Figure 9(a)). The proposed design is reliable, safe, portable, light in weight, and easy to use. The design is based on the concept of coupling the ankle and knee motion together to provide an assistive force to lift the foot during swing phase. The four-bar linkage involves four rigid links attached together at pivoting point. The AFO is attached to the calf and foot of the wearer with link attachment and another curved bar is there in the back of the thigh. When the knee is flexed for 5–20 degrees, the thigh of the wearer contacts the bar. This contact force generates a lifting force through the four-bar linkage action on the ankle. If the knee is not flexed or the ankle is sufficiently flexed this force is not generated. Two springs connect the top link of the four-bar linkage and thigh link. During locomotion one of the springs remains prestressed and another one fully compressed to prevent bouncing and oscillation of the thigh link. The assisting torque at ankle is related to the knee flexion and the authors demonstrated the relation in a graph. The degree of ankle dorsiflexion assistance and timing can be adjusted for individual wearers by changing the length of the links, spring stiffness, and its attachment point.

To restore the legged locomotion of person with spinal cord injury, Polinkovsky et al. [9] developed “insertion point eccentricity controlled” (IPEC) AFO that can provide assisting torque during push-off. The IPEC AFO includes a mechanical brace in addition to functional neuromuscular stimulation (FNS). FNS system restores movement patterns of affected limb by applying electrical stimulation to the controlling nerves of the muscle. It was not possible to restore all the functions of the lower limb through FNS. Moreover, toe-dragging and foot-slap remained as abnormalities. The mechanical system of the IPEC AFO is comprised of a pretensioned spring, a four-bar mechanism, and a crank slider mechanism (Figure 9(b)). The slider mechanism controls the insertion point of the spring and the four-bar mechanism delivers torque to the ankle joint while the spring provides the actuation force. The slider mechanism, affixed to the drive link or back plate, consists of a 6.5 W DC motor integrated with 19.1 ceramic planetary gear head and a 2-channel encoder. The motor, coupled via a timing belt, drives a lead screw which has two turns per inch pitch. Power transmission to the slide mechanism was done by two acme nuts. The spring force, acting on the back plate, was kept perpendicular to the lead screw translating force by means of two 1/4 inch diameter bearings. This mechanism allows the slider to move through the drive link pivot point, and changes the moment arm of the spring and the direction of the torque acting on the ankle. The control system is comprised of a potentiometer at the ankle joint, force sensing resistor under sole, and an encoder on the motor. These sensors help detect the movement of affected limb and the slider can position itself accordingly to provide dorsiflexion assisting torque during swing phase to prevent foot drop. The AFO is capable of providing 3.51 Nm torque in dorsiflexion direction and 3.88 Nm in plantarflexion direction with a spring having initial pretension 77 N and spring modulus 3110 N/m.

### 4.6. AFO with Series Elastic Actuator

Blaya and Herr [24] were the first to use series elastic actuator (SEA) for ankle foot orthosis construction. An SEA actuator in the dorsal part, a rotary potentiometer at ankle, and six capacitive force transducers under the sole were mounted on a standard polypropylene AFO to assist drop-foot gait. The motor of the SEA actuator is controlled by a computer, which takes decisions based on an adaptive control algorithm. The algorithm separated the gait cycle into three different states, which are detected by the signals from the ankle and force sensors. From heel strike to midstance, the author modeled the ankle function as a linear torsional spring, and during this period the SEA adjusts the stiffness to prevent foot-slap. From middle to late stance zero impedance is provided by SEA to allow free ankle motion and in swing phase constant impedance is provided to ensure the toe-clearance.

**Figure 10:**
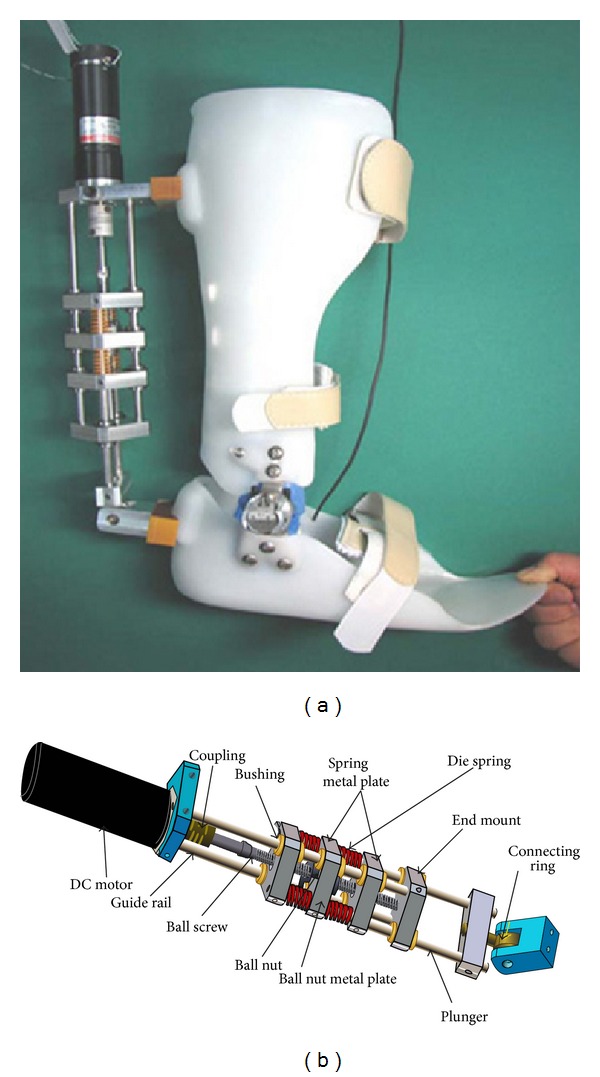
(a) An articulated AFO with SEA, (b) a series elastic actuator [42].

Hwang et al. [42] developed an active ankle foot orthosis that features series elastic actuator (SEA), to prevent drop-foot and foot-slap (Figure 10(a)). The basic structure of the AFO consists of an ankle joint that can allow free plantarflexion/dorsiflexion. The control system of the AFO includes four force sensing resistor (FSR) sensors placed under plantar surface of the foot section of the AFO (heel, hallux, and 1st and 5th metatarsal base position), a rotary potentiometer, and an encoder mounted on the motor of SEA. An off-board master processor detects the phase of the gait using the signals from the sensors and a slave processor controls the motor of the SEA according to the output of the master processor and encoder (position and motor speed data). SEA employs a DC electric motor, four compression springs in series with the motor, ball screw, ball nut, bushing, and some other components (Figure 10(b)). The DC motor drives the ball screw which converts the rotary motion of the ball nut. Depending on the direction of the motor rotation, the ball nut moves front and back on the ball screw. With the motor rotation, the ball nut flanges push on the compression springs. In turn, the compression spring moves the metal plate which is attached to the output plungers. During loading response, the actuator makes the SEA length short and provides plantarflexion motion. From midstance to terminal stance, SEA provides dorsiflexion motion by increasing the length. During preswing the actuator rapidly changes the length by compressing the springs to provide plantarflexion moment around the ankle to assist push-off, and in swing phase SEA makes sure that the ankle joint is dorsiflexed enough to avoid drop-foot.

## 5. Discussion

### 5.1. Design Achievements

The main functions of an AFO for drop-foot prevention are to (a) provide a moderate resistance during loading response to inhibit foot-slap, (b) allow free dorsiflexion in stance phase, and (c) provide large resistance in swing phase to obstruct foot drop. Among these functions all AFOs must possess the third functionality to prevent drop-foot. All the designs reviewed in this paper have at least one of the first two requirements; some of the designs included additional functional features that improved the gait of the patients. These functional features are as follows.

- Adjustability of initial ankle angle at which the heel strikes the ground.
- Adjustability of ankle stiffness.
- Assisting push-off by providing plantarflexion moment.
- Harvesting energy from gait.
- Adjustability to respond to the ground variation.

The passive AFOs presented in this paper prevent drop-foot by providing dorsiflexion assisting moment or direct physical resistance. These designs are capable of improving gait deficiencies, preventing drop-foot, and some of these possess advantages in terms of safety, cost, and compactness. Amidst all the designs described in this paper, only dream brace and AFO with oil damper are commercially available. Successful integration of passive fluid power systems resulted in excellent motion control of the ankle during locomotion. Various literatures have shown that AFO with oil damper increases stability, kinetics, and kinematics of pathological gait [36, 44, 45].

Two of the most promising AFO designs are found to use magnetorheological fluid. MR fluid provides modulation of articulated joint stiffness and better ability to control motion. Although these active devices are lacking the ability to provide assisting torque, these are the most promising designs to be used as daily-wear device [25, 40, 41]. Moreover, one of the AFOs with MR fluid damper facilitates ascending and descending stairs or inclined surface [32]. Series elastic actuators can provide more functionality than any other active elements used in ankle foot orthotic devices. It can provide both plantarflexion and dorsiflexion assisting moment, modulation of ankle joint stiffness, and shock absorption. We have found two similar designs using SEA, which are capable of providing excellent motion control of foot and preventing drop-foot [24, 42]. Both passive and active four-bar linkage mechanisms are incorporated in AFO design to provide dorsiflexion assisting moment; moreover, using plastic, aluminum, or carbon fiber reduced the weight of the device [41, 43].

### 5.2. Remaining Issues and Future Direction

Ankle foot orthosis designs for drop-foot should preferably include all the features just investigated. However, there are some issues to be resolved to develop an effective AFO. All the designs we have discussed, which are the sagittal plane device, have one degree of freedom. Inversion and eversion motion limitation results in discomfort and unnatural gait. Most of the AFOs lock the ankle in a preferred position to ensure ground clearance in swing phase and perturb the free movement of the ankle during walking resulting in excessive knee flexion motion, instability in stance, and elevation of energy cost of walking. Although the incorporation of various passive elements resolved these problems, activation timing and operation of those elements in different phases of gait cycle remained as an issue. Motion control of the passive AFO depends on activation of springs, switches, or valves, which is controlled in an open-loop method as the individuals walk. Moreover, these elements are not capable of adapting in a changing walking condition like stair climbing or descending.

On the other hand, to date, there is no available commercialized or daily-wear active AFO. All the active devices exist only in laboratories and are used for rehabilitation and measurement of physical properties. Tethered power system, bulky and inconvenient size are the negative factors of active AFO for independent walking. Most of the active AFOs use angle measuring sensors and foot sensors to detect the different phases of gait cycle, since they are not capable of sensing unusual situation and that is why these AFOs lack adaptability in varying conditions. Moreover, there is no daily-wear device that addresses plantar flexor muscle deficiencies, which is an important issue for acute ankle injury.

Future research should be conducted to develop untethered active AFO with compact, lightweight actuator that can meet the biomechanical requirements of an impaired person efficiently. A control system should be developed with new type of instruments that can surpass the limitations of current phase detecting sensors to improve adaptability to changed environment and provide independent walking.

## 6. Conclusion

We have conducted a literature review of articulated ankle foot orthosis designed for drop-foot treatment. Recent studies suggest that, for daily-wear application, it is necessary to develop a lightweight, compact, efficient, and untethered AFO that can accommodate to the various functional deficits of the ankle joint within a single device. Active ankle foot orthotic devices possess the potential to be used as daily-wear device as it improves the pathological gait by preventing drop-foot during swing phase, permitting normal ankle motion during other phases, and assisting push-off in some cases. However, there are substantial technological challenges to accommodate all these characteristics within a single device. Understanding the design and mechanism of each AFO is crucial for the development of a new AFO that would enhance walking ability of the individuals and ensure comfort as well.

## Figures and Tables

**Table 1:** Summary of reviewed design.

AFO type	Weight	Active joint element	Locking/ assistive moment mechanism	Maximum resistive moment	Advantage	Disadvantage
Dream brace [33]	0.35 Kg	One-way frictional clutch	Resistive	1.6–1.8 Nm for small and medium size	Light weight, compact	Fixed resistance in plantarflexion direction throughout the gait cycle

AFO with oil damper [27]	0.40 Kg	Oil damper	Resistive	5–20 Nm per 10° of plantarflexion	Adjustability of initial angle and stiffness, lightweight	Resistive torque cannot be modulated

AFO with passive pneumatic element [38]	—	Passive pneumatic element	Resistive	4 Nm	Compact, lightweight, variable motion control	Difficult to adjust the constraint force

University of Illinois AFO [10]	1 Kg	Cam lock mechanism	Locking	—	Energy harvesting capacity, variable motion control	Bulky size, no resistance during loading response

Okayama University AFO [39]	0.86 Kg	Pneumatic actuator	Assistive	2 Nm	Energy harvesting capacity, untethered	Bulky size, small assistive torque

iAFO [40]	1.3 Kg	MR damper	Resistive	5 Nm at 20°/s angular velocity	Different modes of rigidity during gait cycle, smaller power system required, untethered	Heavy, control setting needs skilled physician

AFO with MR brake [25]	1.6 Kg	MR brake	Resistive	24 Nm	Large braking torque, modulation of stiffness in different phases of gait	Heavy, tethered and high energy consuming

Halmstad AFO [32]	—	MR damper	Resistive	—	Responds to change of surface condition, simple and untethered design, only three control parameters	Bulky, not capable of producing assisting torque

AFO with CMRFB [41]	0.99 Kg	Compact MR fluid brake and a spring unit on the ankle joint	Resistive	10 Nm	Lightweight, compact, better motion control	Tethered, complex mechanism

AFO with SEA [24]	2.6 Kg	Series elastic actuator	Assistive	—	Adjustable ankle impedance, provides both plantarflexion and dorsiflexion assistance	High weight, tethered

AAFO with SEA [42]	—	Series elastic actuator	Assistive	—	Plantarflexion and dorsiflexion motion control, adjustable impedance	Bulky size, tethered

AFO with four-bar mechanism [43]	0.625 Kg	Passive four-bar linkage mechanism	Assistive	—	Simple, lightweight, does not restrict motion other than swing phase	Uncomfortable, not adjustable, unable to prevent foot-slap, actuation depends on knee flexion

IPEC AFO [9]	0.46 Kg	Active four-bar linkage and spring mechanism	Assistive	3.51 Nm in dorsiflexion direction and 3.88 Nm in plantarflexion direction	Lightweight, able to provide both plantarflexion and dorsiflexion moments	Bulky, complex mechanism
